# Global status of policies and practices for systematic TB screening in high-burden countries

**DOI:** 10.5588/ijtldopen.25.0434

**Published:** 2025-12-10

**Authors:** A.L. Innes, R. Matji, D. Menzies, K. Rade, J. Alacapa, B. Sanni, E. Qadeer, N. Kak, A. Paydar, N. Kumar, T.T Pakasi, O. Chijoke-Akaniro, M. Ismail, R.A. Fabella, N. Ndjeka, A. Burua, V.L. Dinh, D. Falzon, C.R. Miller

**Affiliations:** 1 Innes Consulting, Hanoi, Vietnam;; 2 AQUITY Innovations, Pretoria, South Africa;; 3 McGill International TB Centre, Montreal Chest Institute, Research Institute of the McGill University Health Centre, Montreal, QC, Canada;; 4 Independent Consultant, Mumbai, India;; 5 Data and Implementation Sciences for Health (DISH), Manila, Philippines;; 6 AQUITY Innovations, Houston, TX, USA;; 7 Health Solutions International, Islamabad, Pakistan;; 8 AQUITY Innovations, Washington, DC, USA;; 9 McGill International TB Centre, Research Institute of the McGill University Health Centre, Montreal, QC, Canada;; 10 Central Tuberculosis Division, Ministry of Health and Family Welfare, New Delhi, India;; 11 Directorate of CDC, Ministry of Health Republic of Indonesia, Jakarta Selatan, Indonesia; 12 National Tuberculosis, Leprosy and Buruli Ulcer Control Programme, Federal Ministry of Health Nigeria, Abuja, Nigeria;; 13 National Tuberculosis Programme, Common Management Unit for AIDS, TB and Malaria, Islamabad, Pakistan;; 14 National Tuberculosis Program, Department of Health, Manila, Philippines;; 15 National Tuberculosis Programme, National Department of Health, Pretoria, South Africa;; 16 Department of Medicine, University of Cape Town, Cape Town, South Africa;; 17 National Tuberculosis and Leprosy Programme, Communicable Disease Prevention and Control, Kampala, Uganda;; 18 Infectious Diseases Institute (IDI), Kampala, Uganda;; 19 National Tuberculosis Program, National Lung Hospital, Hanoi, Vietnam;; 20 Department for HIV, Tuberculosis, Hepatitis and Sexually Transmitted Infections, World Health Organization, Geneva, Switzerland.

**Keywords:** tuberculosis, systematic screening, chest X-rays, diagnosis, imaging, artificial intelligence

## Abstract

**BACKGROUND:**

The global status of policies and practices for systematic TB screening has not been described since the World Health Organization (WHO) guideline update in 2021. In 2024, the WHO Global Programme on Tuberculosis & Lung Health commissioned a questionnaire survey and in-depth reviews of systematic screening for TB disease in high-TB-burden countries.

**METHODS:**

A short-answer and multiple-choice questionnaire was sent to the 30 highest-TB-burden countries to query national policies and the scale of systematic screening, prioritising practices and results among targeted populations. In eight of the 30 countries, mixed-methods in-depth reviews comprised national policy desk reviews; stakeholder interviews; subnational site visits; and analyses of TB cascade of care data from systematic TB screening (2021–2023).

**RESULTS:**

Systematic TB screening showed signs of expansion since 2021 in eight high-TB-burden countries. The questionnaire survey and in-depth reviews identified best practices including chest X-ray prioritisation in parallel with or replacing symptom screening, computer-aided detection for chest X-ray interpretation, machine-learning spatial analytics, and intensive community mobilisation.

**CONCLUSION:**

High-TB-burden countries have expanded systematic TB screening, but national data systems for monitoring and evaluation must be strengthened to evaluate systematic screening results. Funding and human resource mobilisation is critical for progress. Artificial intelligence and innovative tools may improve implementation quality, enhancing existing human workforce capacity in the context of limited resources.

More than 10 million people fall ill with TB each year. In 2023, TB regained its place as the leading cause of death from an infectious disease, overtaking COVID-19.^1^ The World Health Organization (WHO) released the first consolidated guideline on systematic screening for TB disease in 2013.^2^ In the same year, the *International Journal of Tuberculosis and Lung Disease* published a ‘State of the Art’ series on active case finding for TB,^3–9^ addressing important aspects of systematic TB screening including active case finding in high-risk groups, methodological challenges to active case finding evaluation, programmatic approaches, and the benefit of active case finding to individuals and communities. An update to the WHO systematic TB screening guideline in 2021^10^ expanded the recommendation for population-wide systematic screening to settings with a TB prevalence of 0.5% and higher. Computer-aided detection (CAD) artificial intelligence was also recommended for the first time in 2021 as an alternative to human readers for interpreting digital chest radiography (CXR) for TB screening and triage in individuals aged 15 years or older. Additionally, molecular WHO-recommended rapid diagnostics (mWRDs) were recommended in 2021 as a screening tool to improve the accuracy of symptom screening in populations at increased risk for TB. Alongside these guideline updates, the understanding of TB disease classification in recent years shifted from the binary ‘latent’ and ‘active’ TB to a spectrum of disease states^11,12^ that recognises the significance of asymptomatic TB^13^ and has resulted in intensified interest in systematic TB screening.^14,15^

In the context of these developments, countries have been implementing and scaling up systematic TB screening, but the scope and status of implementation have not been described at the global level. This article describes the current status of systematic TB screening in high-burden countries. A WHO-commissioned questionnaire survey and in-depth reviews were conducted in 2024 in high-TB-burden countries, which aimed to summarise the screening tools and algorithms that are used in facility and community settings among a range of populations, prioritising those with increased risk for TB and including the general population in areas with high TB prevalence. In October 2024, a 3-day technical consultation on systematic TB screening was held in Geneva, Switzerland, convening those who conducted the questionnaire survey and the in-depth reviews, civil society representatives, researchers, and funding agencies, as well as national TB programme leaders from the countries where the reviews were done. Here, we describe the best practices and challenges that were identified from the questionnaire survey, the in-depth reviews, and the technical consultation. The knowledge gaps, challenges, and lessons learned in high-TB-burden countries highlight potential future directions for systematic TB screening, with the goal to accelerate progress for ending TB.

## METHODS

From July to September 2024, the WHO Global Programme on Tuberculosis & Lung Health conducted a questionnaire survey of systematic TB screening that gathered information from the 30 countries with the highest burden of TB disease. In-depth reviews on systematic TB screening were also performed in 8 of the 30 high-burden countries (India, Indonesia, Nigeria, Pakistan, Philippines, South Africa, Uganda, and Vietnam) for further insights into the policies, practices, and results of systematic screening implemented from 2021 to 2023.

### Questionnaire survey and in-depth reviews

A short-answer and multiple-choice questionnaire survey (see Supplementary Data) was sent to national TB programmes from the 30 countries with the highest burden of TB. The survey, which was self-completed by each national programme and returned via email to the survey team, probed questions on systematic TB screening in each country including the policies that are in place, screening tools used, and populations screened, as well as the tools and algorithms applied for each population. Some clarifications were requested in follow-up emails, and in the eight high-burden countries conducting in-depth reviews, questionnaire responses were further probed during the site visits and stakeholder meetings. Quantitative results for the TB cascade of care were collected for three targeted populations (people living with human immunodeficiency virus [PLHIV], contacts of people with TB, and people deprived of liberty)^16^ from 2021, 2022, or 2023; results from the most recent year were selected for analysis, since some countries reported results for fewer than 3 years. Quantitative results for these three targeted populations were not summed, since some individuals may have belonged to more than one population or may have been screened more than one time per year.

Eight of the 30 high-burden countries were selected for in-depth reviews based upon known implementation of systematic TB screening conducted since 2021 (or earlier) and for representation of the Western Pacific, Southeast Asia, and Africa regions. The in-depth reviews comprised input from desk reviews of each country’s TB screening policies and guidelines; stakeholder interviews; observations from site visits to subnational regions, states, provinces, and districts; and analysis of the TB cascade of care data from systematic TB screening (2021–2023). The number and location of subnational sites were selected based upon their representativeness of the country’s TB epidemiology as well as the feasibility of completing all site visits within the timeframe of the in-depth reviews (July–September 2024). Qualitative and quantitative data were collected during the in-depth reviews for a comprehensive understanding of how national TB programmes are applying WHO policy guidance and using the available screening and diagnostic tools for systematic TB screening activities. Qualitative data were collected from in-depth interviews using semi-structured questionnaires (Supplementary Data), which were adapted from the questionnaire survey used for the 30 high-burden countries, among purposively selected stakeholders at national and subnational levels. The interviews probed stakeholder and site-level insights on the algorithms used for systematic screening, including the screening and diagnostic confirmatory tests; the populations screened; and the challenges and enablers for screening implementation. Stakeholders included national health programmes, implementing organisations, donor organisations, and professional societies. Data collection tools were translated from English into the local language where needed.

The in-depth reviews collected quantitative TB cascade of care data at national and subnational levels in the eight high-burden countries, including data for prioritised populations with risk factors for TB (PLHIV, contacts, and people deprived of liberty) as well as facility- and community-based systematic screening. When national-level TB cascade data were not available from systematic screening implementation, project-level data were reviewed to inform the country’s systematic screening status. Screening encounters were defined as visits that were conducted specifically for screening purposes (community-based screening) or alongside health facility visits (facility-based screening). Screening encounters in facilities thus represented the number of visits and not necessarily the number of people. The proportion of new and relapse TB cases attributed to systematic TB screening was calculated from data reported by each high-burden country to WHO from 2021 to 2023.^1^ Programmatic data were also used as a reference point for the number of people detected with TB from all types and locations of TB screening.

Ethics review was not necessary for the questionnaire survey or the in-depth reviews. The list of countries whose national TB programmes responded to the questionnaire survey is reported, but the survey findings do not identify individual programmes. All data that were reviewed for the questionnaire survey and the in-depth reviews were de-identified.

## RESULTS

Among the 30 high-TB-burden countries that were contacted for the survey, 24 countries responded, including the five countries with the highest TB incidence. Twenty-two (92%) countries reported using CXR for screening, with 17 (71%) countries using CAD to support CXR interpretation. Systematic screening was recommended by national guidelines in all 24 countries for PLHIV and contacts of people with pulmonary TB disease, and 96% of countries had guidelines for screening people deprived of liberty who reside in prisons and detention centres. Screening was implemented in practice in all 24 countries for PLHIV and in 20 (83%) countries for contacts and people deprived of liberty (Table 1). For contacts, screening by symptoms and CXR was reported by 18 (75%) countries, and only 9 (38%) countries reported screening by symptoms alone. In the countries that participated in the questionnaire survey and provided quantitative data for 2021, 2022, or 2023, there were more than 20 million screening encounters for PLHIV, nearly 8 million for contacts, and over 3 million for people deprived of liberty (Table 1). Among these, TB disease was detected in 157,592 PLHIV, 95,293 contacts, and 29,095 people deprived of liberty.

**Table 1.** Screening practices, methods, and quantitative results for people living with HIV, contacts, and people deprived of liberty in high-TB-burden countries.^**A**^

Systematic TB screening	PLHIV	Contacts	People deprived of liberty
Total countries responding, N	24	24	24
Screening^**B**^			
Recommended, n (%), yes	24 (100)	24 (100)	23 (96)
Implemented, n (%), yes	24 (100)	20 (83)	20 (83)
Annually,^**C**^ n (%), yes	4 (17)	NA	NA
More often,^**C**^ n (%), yes	18 (75)	NA	NA
Screening methods^**B**^^,^^**D**^			
Symptoms only, n (%), yes	15 (63)	9 (38)	8 (33)
Symptoms and CXR, n (%), yes	13 (54)	18 (75)	13 (54)
Includes CRP, n (%), yes	5 (21)	NA	NA
Quantitative results (2021, 2022, or 2023)^**E**^			
Number of screening encounters^**F**^	20,872,029	7,828,626	3,034,915
Number of people detected with TB disease^**G**^	157,592	95,293	29,095

CRP = C-reactive protein; CXR = chest X-ray; N = number; NA = not applicable; PLHIV = people living with HIV.

A The 30 high-burden countries were defined by two criteria: the top 20 countries in terms of the estimated number of new (incident) cases in 2019 plus the 10 countries with the highest TB burden in terms of the incidence rate (new cases per 100,000 population), which are not already in the top 20 and that meet a minimum threshold of 10,000 new TB cases per year.^1^ Of the 30 countries, 24 responded to the questionnaire survey.

B Missing responses were excluded from the calculation of percentages.

C Annual and ‘more often’ screening frequencies only apply to PLHIV and are not applicable to contacts and people deprived of liberty.

D Most countries reported more than one screening method that varied by the site and setting.

E Quantitative results for each country were selected for the most recent year of reporting from 2021, 2022, or 2023 and then summed for PLHIV, contacts, and people deprived of liberty.

F Number of high-burden countries that reported the number of people screened: PLHIV (n = 17), contacts (n = 18), and people deprived of liberty (n = 13).

G Number of high-burden countries that reported the number of people detected with TB disease: PLHIV (n = 18), contacts (n = 16), and people deprived of liberty (n = 19).

The survey for systematic TB screening in facility- and community-based settings showed a range of coverage and screening by CXR across the 24 countries (Table 2). Systematic screening for patients with chronic respiratory disorders was reported by the largest number of countries (22 [92%]), counting both partial and full implementation; 75% of countries reported screening by CXR in this population, which was higher than CXR screening for other facility-based populations such as those with smoking (29%) and substance use disorders (25%). In community settings, only one country reported full implementation of general population-wide systematic screening, while 20 (83%) countries reported partial implementation, which included limited pilot studies or research studies.

**Table 2.** Systematic TB screening in facility and community settings in 24 high-TB-burden countries.

Settings and populations	Systematic TB screening, total countries responding (N = 24)		If systematically screening for TB, by chest X-ray
Facility-based	n (%), partial^**A**^	n (%), full^**A**^	n (%), yes
Chronic respiratory disorders	11 (46)	11 (46)	18 (75)
Diabetes mellitus	10 (42)	11 (46)	11 (46)
Cigarette smoking	7 (29)	6 (25)	7 (29)
Alcohol/drug use disorder	6 (25)	7 (29)	6 (25)
General out-patients and in-patients	6 (25)	12 (50)	8 (33)
Community-based			
Poor urban slums	7 (29)	11 (46)	10 (42)
Homeless	6 (25)	8 (33)	7 (29)
Indigenous/remote communities	11 (46)	6 (25)	9 (38)
General population, population-wide	20 (83)	1 (4)	12 (50)

N = number.

A Partial or full implementation of systematic screening was indicated through multiple-choice responses (‘No’, ‘Yes’, or ‘Yes, partially’). Missing responses were excluded from the calculation of percentages.

The in-depth reviews provided further insights into how systematic TB screening was conducted at national, subnational, and site levels in eight high-burden countries. Since 2021, systematic TB screening showed signs of expansion in most countries in terms of screening encounters from all strategies combined (Table 3). Increased use of mWRDs and engagement of non-TB private and public facilities were described as significant contributions to TB notifications in some settings. In 2023, TB treatment coverage ranged from 57.4% in Vietnam to 90.1% in Uganda (Table 3), while the proportion of bacteriologically confirmed pulmonary TB among total TB notifications was highest for Nigeria (82.6%) and Vietnam (82.5%) and lowest for Indonesia (52.9%), Pakistan (50.4%), and the Philippines (44.3%). In 2023, the proportion of new and relapse notified TB cases that originated from systematic screening in these eight countries ranged between 0.63% and 48.08%.^1^ Screening encounters included community- and facility-based activities; reporting for facility systematic screening did not differentiate between symptom-based and CXR-based algorithms in all settings.

**Table 3.** Country population, TB epidemiology^**A**^ (2023), and systematic screening progress (2021–2023) in eight high-TB-burden countries.

Indicators	India	Indonesia	Nigeria	Pakistan	Philippines	South Africa	Uganda	Vietnam
Population (2023) (million)	1,438	281	228	248	115	63	49	100
TB incidence rate per 100,000 population (2023)	195	387	219	277	643	427	198	182
TB incidence number (2023)	2,800,000	1,090,000	499,000	686,000	739,000	270,000	96,000	182,000
TB treatment coverage rate (2023)	85.1%	73.8%	73.6%	69.4%	77.9%	78.5%	90.1%	57.4%
Proportion of bacteriologically confirmed pulmonary TB out of all notified cases (2023)	62.2%	52.9%	82.6%	50.4%	44.3%	67.0%	67.9%	82.5%
Total number of screening encounters^**B**^								
2021	280,000,000	303,160	4,421,170	62,849,381	1,131,542	78,009,082	21,426,060	NA
2022	262,000,000	774,102	9,065,480	75,678,190	2,105,198	87,529,452	30,713,527	889,821
2023	238,000,000	1,282,305	3,845,167	80,664,378	5,853,855	86,932,268	32,975,433	1,393,977
Proportion of new and relapse TB cases notified and detected through screening (2023)^**A**^	34.72%	0.63%	5.17%	48.08%	5.33%	NA	20.44%	30.07%

NA = not available.

A TB epidemiology data (2023) and the proportion of new and relapse TB cases notified and detected through screening (2023) were obtained from the World Health Organization Global Tuberculosis Report 2024 country profiles.^1^

B Screening encounters (2021–2023) were reported during the in-depth reviews and were defined as numbers of visits conducted for systematic screening. There is high likelihood of variation across different countries due to how screening encounters were defined and reported. Screening encounters for 3 years were reported for all countries except Vietnam, for which the systematic screening data (community- and facility-based targeted populations) were reported using a system designed for systematic screening starting in 2022.

Since the WHO’s 2021 guideline update, the prioritised placement of CXRs was one of the most significant changes to systematic TB screening algorithms in the eight high-burden countries. The most common algorithm reported was parallel screening with CXR and symptoms, although all countries described variability with implementation due to CXR accessibility (i.e., if CXR was not available, single screening with symptoms was done). Limited access to CXR in some settings led to symptom screening in health facilities for all eight countries, as was described during the in-depth reviews and is consistent with the questionnaire survey findings (Table 2). The in-depth reviews also found that most countries were in the process of transitioning to a symptom-agnostic screening algorithm, and that the success of this transition was impacted by the availability of CXR and the quality of CXR interpretation.

## DISCUSSION

The survey and the in-depth reviews identified best practices and challenges from systematic TB screening in high-burden countries.

### Best practices for systematic TB screening

The eight high-burden countries evaluated for the in-depth reviews currently implement screening among targeted populations at risk for TB in communities and facilities, while population-wide mass screening is implemented as pilots or research in most settings. The countries are deploying a variety of innovative approaches for screening (Table 4). Vietnam’s ‘Double X’ algorithm prioritises CXRs and mWRDs to improve the accuracy of TB disease detection for targeted screening in community and facility settings.^17^ South Africa’s **T**argeted **U**niversal **T**est and **T**reat (TUTT) algorithm uses mWRDs as the first test to screen for TB disease among TUTT risk groups or symptomatic individuals, while asymptomatic individuals are screened first with CXR and then referred for mWRD testing if CXRs are TB-presumptive.^18^ In Uganda, a decline in TB notifications post COVID-19 pandemic led to the **C**ommunity **A**wareness, **S**creening, **T**reatment, and Prevention of TB and Leprosy (CAST) initiative in 2021, which increased TB notifications at the national level.^19^ CAST implementation further accelerated the increase in TB notifications from community mobilisation that was reported before the pandemic (2016–2019).^20^ Nigeria’s high-yield community-based systematic TB screening employs **W**ellness **o**n **W**heels (WoW) trucks equipped with digital CXR and CAD^21,22^ as well as CAD-enabled ultraportable digital CXRs.^23^ Machine-learning spatial analytics have the potential to guide community-based systematic TB screening, currently being evaluated in Pakistan’s MATCH-AI (**M**apping and **A**nalysis for **T**ailored disease **C**ontrol and **H**ealth system strengthening-**A**rtificial **I**ntelligence) algorithm in the SPOT-TB study that began enrolment in 2024.^24^ Machine-learning spatial analytics have also been piloted in South Africa, Nigeria,^25^ and the Philippines. India is utilising vulnerability mapping to guide community screening, wherein a risk-scoring system prioritises further testing for those at highest risk for TB.

**Table 4.** Best practice examples of systematic TB screening from eight high-TB-burden countries.

Screening model (country)	Population and setting	Screening test	Diagnostic test	Distinctive feature
CAST (Uganda)	Household and close contacts and targeted hotspots for population-wide screening	Signs and symptoms of TB	mWRD	Intensive community awareness and sensitisation
Double X (Vietnam)	Contacts and other risk groups	CXR with CAD in parallel with symptoms	mWRD	Screening for TB disease is integrated with testing for TB infection for close contacts and people deprived of liberty
	Facility- and community-based screening for targeted populations			
TUTT^**A**^ (South Africa)	Facility-based screening of TUTT groups, people with TB symptoms, and people in high-risk groups^**B**^ who have TB-presumptive CXRs	•TUTT groups (with or without symptoms) → mWRD		mWRD as the screening/diagnostic test for TUTT groups and people with TB symptoms
		•Symptomatic people (with or without risks) → mWRD		
		•Asymptomatic (among high-risk groups) → CXR	mWRD	mWRD as diagnostic test for TB-presumptive CXR
Ultraportable digital CXR with CAD (Multiple countries)	Community populations (targeted by TB risks or by spatial analytics)	Varies by setting; ideally parallel screening by CXR and symptoms	Varies by setting; ideally mWRD	Increased access to digital CXR that is equipped with CAD to improve the quality of interpretation
Vulnerability mapping (India)	Community populations (targeted by vulnerabilities)	TB symptoms or CXR	mWRD	Risk-scoring system for vulnerabilities
WoW Truck (Nigeria)	Community populations (targeted by TB risks)	CXR with CAD, chronic cough	mWRD	Early notification of CAD interpretation of CXR results

CAD = computer-aided detection; CAST = community awareness, screening, treatment, and prevention of TB and leprosy; CXR = chest X-ray; Double X = chest X-ray and GeneXpert algorithm; mWRD = molecular WHO-recommended rapid diagnostic; TUTT = Targeted Universal Test and Treat; WoW = Wellness on Wheels.

A TUTT risk groups comprise people living with HIV, contacts of people with TB, and people previously treated for TB disease within the past 2 years.

B High-risk groups comprise the TUTT populations plus people who currently or previously worked in mines and those aged 65 and older.

The best-practice systematic TB screening models (Table 4) consistently place CXRs high in the screening algorithm, often in parallel with symptoms, and underscore the importance of mWRDs as the first diagnostic test for all those who screen positive. However, access to both CXR and mWRD is not guaranteed in all settings, introducing variability in implementation and results. Community awareness and engagement have played a key role in the success of Uganda’s CAST initiative^19^; the Philippines’s barangay health workers^26^; India’s Accredited Social Health Activists (ASHA)^27,28^ and community health workers; and Pakistan’s Lady Health Workers.^29^

### Evaluating the spectrum of TB disease

Due to the expansion of CXR screening, high-burden countries are detecting TB disease among individuals who have no symptoms or who have mild symptoms, known as asymptomatic TB disease (previously called subclinical TB).^13^ TB disease that is diagnosed without bacteriological confirmation (clinically diagnosed TB) is based on clinical examination and a provider’s decision to treat for TB disease, often characterised by symptoms and/or CXR abnormalities.^13^ The accuracy of CXR interpretation is particularly important for detecting asymptomatic TB^16^ and bacteriologically unconfirmed TB disease, although standard protocols to detect these TB disease subgroups are not available in most high-burden countries, as reported during the in-depth reviews. The integration of CAD artificial intelligence into TB screening algorithms to improve the accuracy of CXR interpretation is one of the main innovations introduced since 2021, both from a policy and implementation perspective. CAD-enabled CXR is used, mostly at pilot scale at the time of the in-depth reviews, for community-based systematic TB screening in all eight countries and for 17 (71%) of the 24 high-burden countries that completed the questionnaire survey. The use of CAD and ultraportable CXR decentralises access to high-quality digital radiography^30^ that is critical for increased population coverage.

The survey and the in-depth reviews described the status of high-burden countries’ guidelines and practices for integrating screening for TB disease with TB infection. In these countries, integrating TB screening with testing for TB infection is currently prioritised for contacts and people deprived of liberty. While there are known challenges for diagnosing TB infection,^31–34^ the integration with TB disease evaluation can save time and costs because one interaction with the targeted populations enables screening and testing for both TB disease and TB infection.^35^ Using this integrated approach, CXRs can be obtained with the dual purpose of ruling in or ruling out radiographic abnormalities suggesting TB, followed by diagnostic confirmatory testing for TB disease or evaluation for TB infection, depending upon the CXR result and symptom screen. CAD interpretation of CXR can support the joint evaluation of TB disease and TB infection, although using CAD to rule out TB disease for TB preventive treatment eligibility is limited in practice.

### Tackling resource limitations to support systematic TB screening

Lack of sufficient funding, which is often dependent upon external donors, was reported by all eight countries from the in-depth reviews as one of the biggest challenges threatening continued implementation and scale-up of systematic TB screening. Among the 24 high-burden countries that responded to the survey, almost all rely heavily on donor funding for systematic screening. Following completion of the survey and in-depth reviews, the donor funding landscape has shifted considerably due to the vast reduction of funding from the United States Agency for International Development in March 2025.^36^ The sudden and substantial decrease in donor funding threatens the expansion, and even the continuation, of the best practice models described here and further constrains the scale-up of population coverage by population-wide screening. Limited human resources, notably for trained radiographers to operate ultraportable CXRs, were reported in many of the eight countries from the in-depth reviews. In addition, while WHO guidelines allow CAD to replace human readers for interpreting CXRs among individuals 15 years or older, many settings still require human readers alongside or following CAD interpretation. The diagnostic network capacity in many high-burden countries is insufficient for full scale-up of mWRDs as the first test, which results in continued dependence on smear microscopy and/or clinical symptoms and risk factors. Among the prioritised targeted populations in the eight countries from the in-depth reviews, screening in miners is especially limited, while screening among contacts of all ages has improved but remains suboptimal.

### Optimising yield and population coverage of systematic TB screening

The screening coverage among targeted populations, the diagnostic yields for different screening models, and the proportion of bacteriologically unconfirmed TB varied widely among the eight countries conducting in-depth reviews, limiting the analysis and interpretation of results. The in-depth reviews found that national guidelines are often administered through a decentralised approach, with local funding required for implementation and standard operating procedures that are adapted from national guidelines to the local setting. The variability in implementation across a country’s geographic regions, screening models, and populations challenges the interpretation of screening yields.

Facility-based systematic TB screening often has a high yield in TB detection. This was found in the cascade of care data from the in-depth reviews and is consistent with other studies.^37,38^ Despite being high yield, facility-based systematic TB screening does not always lead to an increase in total notifications, suggesting that some notifications from facility systematic TB screening may be earlier than, not additional to, passive notifications. Nevertheless, a general principle of systematic screening is that earlier diagnosis, even without an increase in total notifications, may provide individual^39^ and public health benefits that reduce TB morbidity and community transmission. The optimal design for community-based systematic TB screening is not clear, in terms of targeted population versus population-wide screening^40^; furthermore, the timeframe evaluated by the in-depth reviews (2021–2023) included the COVID-19 pandemic, confounding the interpretation of TB epidemiological trends. The in-depth reviews found that community-based systematic TB screening has traditionally centred around mobile CXR vans and are often large-scale and costly and require intensive engagement of national TB programmes, all of which limit the frequency and population coverage of implementation.

### Maximising CXR with CAD by increased access, optimised performance, and follow-up

The in-depth reviews found that the availability of digital CXR and CAD equipment is insufficient to enable access to high-quality CXRs for all vulnerable populations. Regulatory approval for mobile and ultraportable CXRs and CAD can be delayed. The variable quality of CXR interpretation without CAD is a well-known challenge.^41^ CAD threshold score selection and calibration are challenging to perform, and optimal thresholds can vary within a country depending on the geography and targeted population; these challenges have been well described.^42,43^ When using CXRs to screen for TB disease, a large proportion of individuals will have TB-presumptive CXRs but negative diagnostic confirmatory testing. Some of these individuals may develop TB disease in the years following the screening evaluation^22,44–46^; other individuals may have infectious or non-infectious diseases that are not TB,^47,48^ and they should be linked to diagnostic evaluation and care. None of the eight countries from the in-depth reviews systematically repeat the evaluation for TB disease among all individuals with TB-presumptive CXRs and negative diagnostic test results.

### Improving interoperability of data systems for systematic TB screening

The proliferation of online, electronic, and case-based data systems for TB in high-TB-burden countries is advantageous overall for TB programming while simultaneously introducing challenges for interoperability across platforms.^49,50^ The in-depth reviews found that TB surveillance systems do not always integrate well with CAD data and dashboards or with one-off systems developed for screening projects. Multi-disease integrated screening is even more complex, since other disease programmes often have their own data systems and requirements. Case-based TB surveillance platforms capture data for individuals at the time of diagnosis for TB disease, while individuals who are eligible for screening or who have been screened (but not yet diagnosed) are not captured in most countries, limiting the accuracy of TB detection yield. Finally, national TB programmes are tracking systematic screening at the national level while many activities are implemented by different partners across different sites.

### Ensuring access to TB detection, care, and cure for vulnerable populations

TB-related stigma, a common challenge identified in the in-depth reviews, hinders engagement of vulnerable populations and communities for TB screening.^51–53^ At the same time, the awareness and capacity to combat stigma among providers is often limited, as expressed during the reviews’ stakeholder meetings. The proportion of male and female vulnerable populations reached and screened for TB differed across settings as observed in programmatic data from the in-depth reviews, which could be due to differences in access and/or awareness and requires a customised approach to address barriers.^54^ Globally, TB notifications are higher for men, but the treatment coverage gap, that is, the ‘missing cases’, is also higher for men,^1^ underscoring the challenges. Finally, engaging people in care who have asymptomatic TB disease may be difficult^55^ and requires community sensitisation as well as effective pre-screen and pre-test counselling. Continuous support from diagnosis through treatment completion is needed for people who are diagnosed with TB disease through systematic screening.

### The future of systematic TB screening

Knowledge gaps were identified during the WHO-commissioned survey, the in-depth reviews of systematic TB screening, and the WHO technical consultation, which may inform future directions.

1. *How should targeted screening and population-wide mass screening be deployed and what population groups and coverage rates would benefit from each approach*? High-TB-burden countries have variations in TB prevalence and notifications by geographic regions and populations. The optimal approach for systematic TB screening is not yet defined across these settings, although current practices fall along a continuum (Figure). More research and modelling studies are needed to guide the selection of screening strategies for optimal results.
2. *What is the most effective strategy to mobilise human resources for increased population screening coverage?* Human resource limitations are one of the most critical barriers to expanding population coverage, and identifying pragmatic and sustainable solutions should be prioritised. CAD-enabled ultraportable CXRs have the potential to effectively decentralise community systematic TB screening and task-shift responsibilities from human experts to CAD software. Training and certificate programmes for community and lay workers to operate CXR and CAD may mitigate some workforce limitations but require buy-in from the local setting.
3. *Can artificial intelligence solutions progress from pilot to programmatic expansion for broader impact?* Artificial intelligence tools such as CAD for CXR interpretation and spatial analytics to map TB disease have potential but remain in the pilot stage of implementation in many high-burden settings. CAD threshold score selection and calibration are complex, and the methodology should be simplified from research protocols to match routine, programmatic expertise. Early results suggest that machine-learning spatial analytics can guide systematic TB screening and improve case detection yields, but more evidence is needed. A strategy deploying ‘smart systematic screening’ that fully leverages technological advances like artificial intelligence may help bring implementation to scale.
4. *Do treatment outcomes differ for TB disease subgroups (asymptomatic or symptomatic) or for those who are screened and diagnosed through active and intensified systematic screening?* Some studies have shown that TB treatment outcomes are worse among those who are diagnosed through active than routine passive case finding,^56,57^ while others show no difference in treatment outcomes.^39^ With this in mind, countries should ensure that a robust system is in place to support people who are initiated on treatment from systematic TB screening in community and facility settings (e.g., active and intensified case finding). This should be prioritised alongside expanding systematic TB screening. Linkage to treatment was found to be no different between asymptomatic and symptomatic individuals with bacteriologically confirmed TB in one study,^58^ and a scoping review^59^ found that treatment outcomes did not differ for people with asymptomatic versus symptomatic TB disease, although the heterogeneity among the 71 studies was a limitation. Currently, treatment regimens are the same for asymptomatic and symptomatic TB disease, but a shorter regimen may be sufficient to cure asymptomatic TB disease. Until such a regimen is identified, the same treatment for TB disease should be offered regardless of symptoms.

**Figure.**
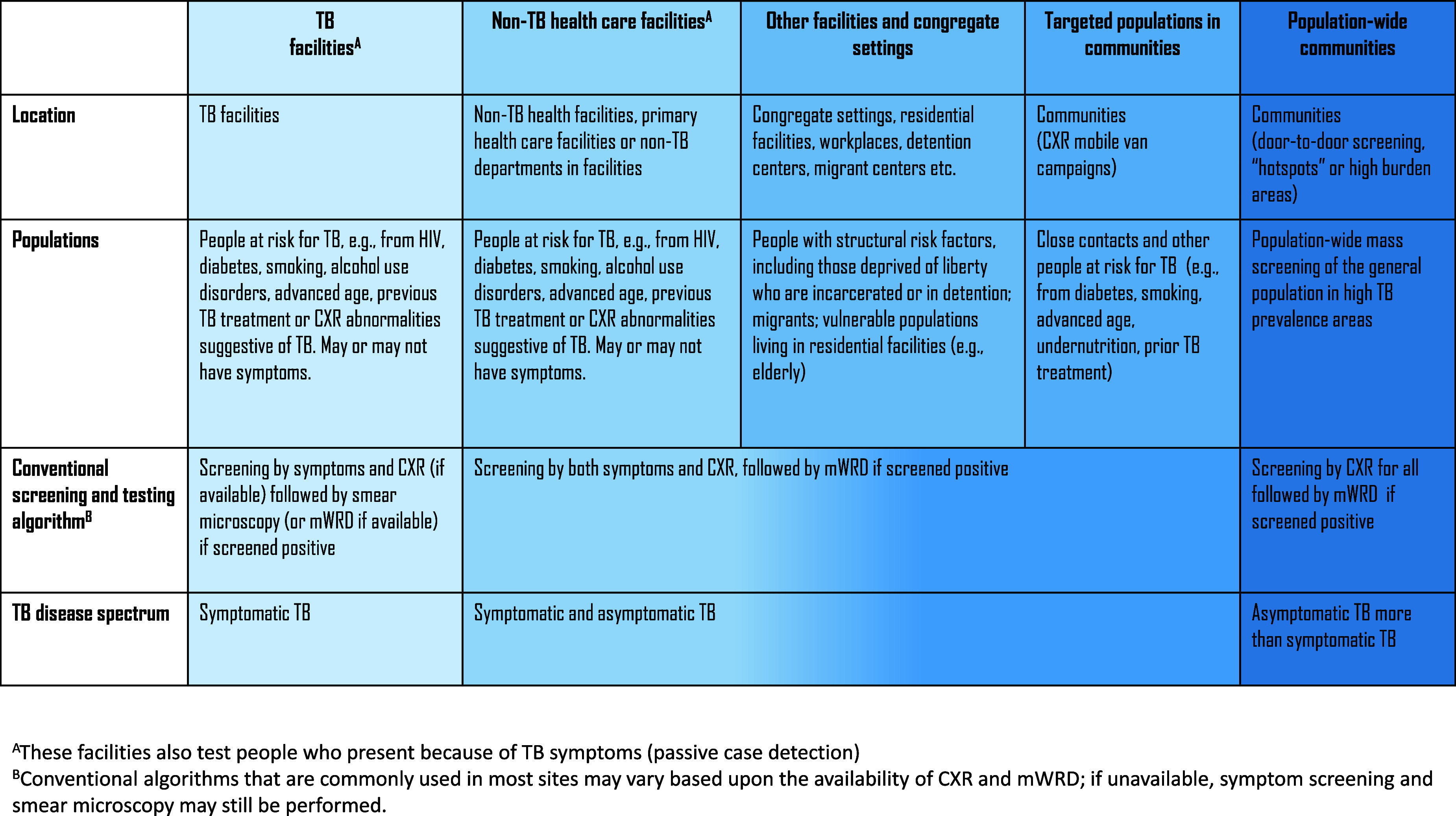
Continuum of systematic TB screening. CXR = chest X-ray; mWRD = molecular WHO-recommended rapid diagnostic.

Lessons from the past decade of programmatic implementation of systematic TB screening can inform effective strategies for the future. The immediate goal is to improve uptake of existing recommendations, such as increasing the use of mWRDs as the first diagnostic test^60^ across the full continuum of systematic TB screening in facility and community settings. Globally, among those who are diagnosed with TB disease, only 48% are tested first with an mWRD,^1^ which may be due to multiple factors including limited funding, suboptimal diagnostic networks, and inaccurate reporting of laboratory data to national TB programmes. Guided by local epidemiology, the continuum of systematic TB screening should include implementation of population-wide mass screening^10,15^ (Figure), potentially using ultraportable CXR and CAD to task-shift responsibilities to workers with less technical expertise.^61–63^ The power of technology has the potential to improve performance across the TB cascade, with additional innovations on the horizon such as machine-learning analysis of cough and lung sounds to complement or even replace CXR screening in the future.^64^ Diagnostic tools that are independent of sputum bacteriological confirmation are also in the pipeline.^65–67^ These innovations may revolutionise the ability to screen and test for TB by mitigating critical barriers affecting implementation.

Our study has some limitations. Six of the 30 high-TB-burden countries did not respond to the survey, but among the 24 that did respond were the five countries with the highest TB burden. In addition, it was only feasible to conduct the in-depth reviews in 8 of the 30 high-burden-countries, although these eight countries did have broad geographical representation and large populations. Self-reported qualitative data may have been biased towards socially desirable responses (e.g., high reported use of CXR may overestimate actual implementation limited by equipment availability and functionality), but responses were cross-checked to some degree during the in-depth reviews in eight countries. Screening encounters that were reported for the in-depth reviews could not be deduplicated in all settings, particularly for facility-based screening, and thus represented the number of visits and not necessarily the number of people screened; this may explain the high number of screenings compared with the national population. Moreover, the survey’s quantitative results for screening encounters among PLHIV, contacts, and people deprived of liberty may overlap among the three populations if individuals belonged to more than one risk group. Given these uncertainties about the accuracy of the denominator data, we did not calculate estimates of yield in this study. Additionally, the types of screening encounters may have varied among settings and included a mixture of people with and without signs and symptoms of TB, who were evaluated along the continuum of systematic screening. Countries’ reporting systems for systematic screening also had limited capacity to distinguish between TB diagnoses by the type of screening. To address this, some countries are developing systems to report systematic TB screening data in addition to routine surveillance (e.g., Vietnam) as well as collecting case-based screening data for contacts (e.g., India, Indonesia, and Vietnam), although more progress is needed.

## CONCLUSION

There is progress on systematic TB screening in high-burden countries. There is less evidence for epidemiological impact, which likely requires increased population screening and testing coverage, as well as improved measurement of effect. Greater impact may be possible through a combination of systematic TB screening in targeted or population-wide communities and facility-based activities, alongside strengthened information systems. Reporting systems for systematic TB screening must be improved to accurately monitor implementation and to inform policies and practices. All approaches require adequate funding and human resources to sustain progress. At the same time, leveraging innovative technology such as CAD for CXR and other artificial intelligence solutions may improve the quality of implementation while enhancing existing human workforces. Accelerating high-quality implementation in the context of limited resources will be necessary to ensure progress towards ending TB.

## Supplementary Material


