# Supplementary material for: Global status of policies and practices for systematic TB screening in high-burden countries
Source: IJTLD Open. 2025 Dec 10;2(12):705–15. doi: 10.5588/ijtldopen.25.0434 (PMC12699962; doi:10.5588/ijtldopen.25.0434)
Supplement: Supplementary file 1 [file ijtldopen25-0434_supplementarydata1.pdf]

1. Interview Guide for National Tuberculosis Programmes:

Survey of policies, activities, and results of TB screening activities

The following interview guide has been developed to gather information from National TB Programmes or other Ministry of Health programmes on current tuberculosis (TB) disease systematic screening activities in key high TB burden countries. The findings will be compiled into a written report and used as a supportive document for a WHO technical consultation on TB screening which will be held in Geneva in October 2024. Thank you in advance for your time.

<u>SECTION 1: General Information about TB screening policy and implementation</u>

Brief questions to identify your programme.	
Date survey completed (dd/mm/yyyy):	
What is your name, and email address (in case clarification is needed)?	
Country	
Name of TB Programme/Institution	
What jurisdictions are covered by your TB programme (municipal, province/state, national)?	
What is the total population served by your TB programme (in millions)?	

National guidelines and policies on TB screening

- Is there a national TB programme guideline for the systematic screening of TB disease?
  - ☐ Yes, a unified guideline (if YES, please attach or provide link)
  - ☐ No single guideline, but some recommendations for screening within guidance for migrants, hospitals, HIV care, etc. (please attach or provide link)
  - ☐ No

- Is screening for TB disease linked to TB preventive treatment (TPT) in your country?
  - ☐ Yes, this is in our guidelines and is being implemented
  - ☐ Yes, this is in our guidelines but is not being implemented
  - ☐ No

- How are national policies for TB screening established? Are any tools (eg ***ScreenTB*** or others) used for strategic planning and developing TB screening policies?

- How have national policies on TB screening changed in the last 3 years? Why?

Funding for TB screening

- How are screening activities funded?
    - ☐ Government (national) funded
    - ☐ Government (local) funded
    - ☐ Donor funded
    - ☐ Special projects
    - ☐ Not applicable
    - ☐ Other (please specify):

- Are screening services always provided free of charge or are there sometimes fees for patients?

Implementation of screening

- Who are the partners implementing TB screening in your country? (include all that apply)
    - ☐ National health programmes (NTP, HIV, MCH, primary care/NCD screening)
    - ☐ Occupational health screening
    - ☐ Penitentiary screening
    - ☐ Implementing partners (INGOs, NGOs, local implementing partners, faith-based organizations, others as applicable)
        - Please specify
    - ☐ Private sector providers (if implementing screening)
    - ☐ Mining sector (if applicable)
    - ☐ Other (please specify):

- How is training prior to screening implementation conducted? Who are the trainers?

- How do screening activities impact the workload and workflow in laboratories?

Overall challenges, enablers

- What are the most challenging aspects of implementing screening?

- What are the biggest challenges with implementing CXR-based screening (with or without computer-aided detection, or CAD)?

- What are enablers for screening implementation? What have you found essential to successful implementation of TB screening?

- In what areas of screening implementation is there a need for further research, guidance, technical support?

Section 2: Tools used for TB disease screening:

Is **chest X-ray (CXR)** use recommended in your national guideline for the screening of TB disease?		☐ Yes<br>☐ No
Is CXR used in practice for the screening of TB disease?	☐ Yes always/regardless of symptoms<br>☐ Yes, sometimes<br>☐ Yes but only after symptom screening<br>☐ No<br>If CXR is **not** used systematically or at all, what are the reasons?<br>☐ Lack of availability of technology<br>☐ High cost<br>☐ Lack of expertise in interpretation<br>☐ Not the preferred screening approach<br>☐ Not determined necessary<br>☐ Other (please describe)	
Is computer-aided detection of TB-related abnormalities on chest radiography (CAD) use recommended in your national guideline for the screening of TB disease?<br>☐ Yes ☐ No		
Is CAD used in practice for interpretation of CXR for the screening of TB disease?	☐ Yes (if **yes**, please describe CAD solution providers)<br>☐ No<br>If yes, how was the threshold to be used for TB disease selected?<br>☐ Used the manufacturer's recommended threshold<br>☐ Pragmatic approach to threshold selection<br>☐ Conducted a calibration study (via operational research)<br>☐ Other (please describe)<br><br>If CAD is **not** used systematically or not at all, what are the reasons?<br>☐ High cost<br>☐ Lack of technical expertise<br>☐ Technological infrastructure (eg internet access, electricity)<br>☐ Shortage of chest x-ray capacity<br>☐ Not necessary (no shortage of human capacity for interpretation)<br>☐ Not approved in country<br>☐ Other (please describe)	

Is use of **molecular WHO-recommended rapid diagnostic test (mWRDs)** recommended in your national guideline to screen people with HIV or other risk groups, regardless of symptoms? (i.e. specifically for *screening* for TB disease, and **not** diagnosis)	☐ Yes<br>☐ No

Are mWRDs used in practice for the **screening** of TB disease (not just for diagnosis)?	☐ Yes, used for screening<br>☐ Not used for screening (may be used for diagnosis)<br><br>If they are **not** used what are the reasons?<br><br>☐ Not aware of option for use for screening<br>☐ Difficulties to implement test in the screening context<br>☐ Financially unfeasible<br>☐ Lack or shortage of mWRD infrastructure or supplies<br>☐ Lack of sample transportation infrastructure<br>☐ Lack of ability to store samples<br>☐ Other (please describe)

SECTION 3: TB DISEASE SCREENING ACTIVITIES IN THREE SPECIFIC HIGH RISK POPULATIONS

I. People living with HIV (PLHIV)

For **PLHIV**, is systematic screening recommended according to your national guideline?	☐ Yes<br>☐ No
For **PLHIV**, is systematic screening implemented in practice?	☐ Yes<br>☐ Yes, partially implemented<br>☐ No
If **yes**, for **PLHIV,** which of the following algorithms are employed for screening? Please refer to this link if needed.	
☐ WHO-recommended four-symptom screen (W4SS) single screening algorithm<br>☐ C-reactive protein (CRP) single screening algorithm<br>☐ CXR single screening algorithm<br>☐ Parallel screening algorithm with W4SS and CRP<br>☐ Sequential positive screening algorithm with W4SS and CRP<br>☐ Sequential negative screening algorithm with W4SS and CRP<br>☐ Parallel screening algorithm with W4SS and CXR<br>☐ Sequential positive screening algorithm with W4SS and CXR<br>☐ Sequential negative screening algorithm with W4SS and CXR<br>☐ mWRD single screening algorithm<br>☐ Other (please describe)	
If yes, with what frequency are PLHIV screened?<br>☐ Once a year ☐ At every visit to a health center<br>☐ Other, or variable (please describe)	

II. Household contacts and other close contacts of individuals with TB disease

For **close contacts**, is systematic screening for TB disease recommended according to your national guideline?	☐ Yes<br>☐ No
For **close contacts**, is systematic screening implemented in practice?<br>*(If **yes**, please also complete next 3 questions)*	☐ Yes<br>☐ Yes, partially

	☐ No
If screening is not being done systematically in all age groups, why not?	
Is systematic screening implemented in all age groups? ☐ Yes ☐ No<br>If **no**, please specify what ages are screened: *(check all that apply)*<br>☐ Individuals aged ≥15 years ☐ Children aged 5-14 years ☐ Children aged <5 years	
For **close contacts,** which of the following algorithms are employed for screening? Please refer to **this link** if needed.	
☐ Screening with cough<br>☐ Parallel screening with cough and CXR<br>☐ Sequential positive serial screening with cough and CXR<br>☐ Sequential negative serial screening with cough and CXR<br>☐ Screening with any TB symptom<br>☐ Parallel screening with any TB symptom and CXR<br>☐ Sequential positive serial screening with any TB symptom and CXR<br>☐ Sequential negative serial screening with any TB symptom and CXR<br>☐ Screening with CXR followed by mWRD<br>☐ Screening with mWRD followed by diagnostic exam<br>☐ Other (please describe)	
For close contacts, what methods are used to reach contacts for screening? (check all that apply)<br>☐ Invite contacts to come to clinic for evaluation<br>☐ Visit the home to screen contacts<br>☐ Other(please describe)	

III. People in prisons and penitentiary institutions

For **incarcerated individuals**, is systematic screening for TB disease recommended according to your national guideline?	☐ Yes<br>☐ No
For **incarcerated individuals**, is systematic screening for TB disease implemented in practice? *(If yes, please also complete next 3 questions)*	☐ Yes<br>☐ Yes, partially<br>☐ No
For **incarcerated individuals,** which of the following algorithms are employed for screening? Please refer to **this link** if needed.	
☐ Screening with cough<br>☐ Parallel screening with cough and CXR<br>☐ Sequential positive serial screening with cough and CXR<br>☐ Sequential negative serial screening with cough and CXR<br>☐ Screening with any TB symptom<br>☐ Parallel screening with any TB symptom and CXR<br>☐ Sequential positive serial screening with any TB symptom and CXR<br>☐ Sequential negative serial screening with any TB symptom and CXR<br>☐ Screening with CXR followed by mWRD<br>☐ Screening with mWRD followed by diagnostic exam<br>☐ Other (please describe)	

SECTION 4: FACILITY BASED TB DISEASE SCREENING ACTIVITIES IN AT-RISK GROUPS

Please indicate if systematic screening for TB disease is performed in hospitals (outpatient or inpatient departments) or primary care centres, for persons with the following risk factors:

**At-risk Groups**	**Systematic Screening *Implemented***	**If screening is implemented, is chest X-ray used?**
People with chronic respiratory disease or lung conditions (including fibrotic lesions on CXR, previous treatment for TB, others)	☐Yes<br>☐Yes, partially<br>☐No	☐Yes<br>☐No
If yes, what algorithm is used for screening this risk group?		
People with diabetes mellitus	☐Yes<br>☐Yes, partially<br>☐No	☐Yes<br>☐No
If yes, what algorithm is used for screening this risk group?		
People who smoke (defined as those who have smoked 100 cigarettes in their lifetime and who currently smoke cigarettes)	☐Yes<br>☐Yes, partially<br>☐No	☐Yes<br>☐No
If yes, what algorithm is used for screening this risk group?		
People who are undernourished, or with a body mass index <18	☐Yes<br>☐Yes, partially<br>☐No	☐Yes<br>☐No
If yes, what algorithm is used for screening this risk group?		
People with alcohol or drug use disorder	☐Yes<br>☐Yes, partially<br>☐No	☐Yes<br>☐No
If yes, what algorithm is used for screening this risk group?		
General outpatients and inpatients (where prevalence of both TB and of TB risk factors is high)	☐Yes<br>☐Yes, partially<br>☐No	☐Yes<br>☐No
If yes, what algorithm is used for screening this risk group?		
Children other than contacts or HIV infection	☐Yes<br>☐Yes, partially<br>☐No	☐Yes<br>☐No
If yes, what algorithm is used for screening this risk group?		
Persons in mental health institutions or clinics	☐Yes<br>☐Yes, partially<br>☐No	☐Yes<br>☐No
If yes, what algorithm is used for screening this risk group?		
Persons in other residential facilities (long term care)	☐Yes<br>☐Yes, partially	☐Yes<br>☐No

	☐No	
If yes, what algorithm is used for screening this risk group?		
Other (please describe risk group and algorithm used)	☐Yes<br>☐Yes, partially<br>☐No	☐Yes<br>☐No

SECTION 5: OTHER LOCATION-BASED TB DISEASE SCREENING ACTIVITIES IN AT-RISK GROUPS

Are there any screening activities in ***community sites*** for the following population groups?

**At-Risk Groups**	**Systematic Screening *Implemented***	**If screening is implemented, is chest X-ray used?**
Poor urban (slum) communities	☐Yes<br>☐Yes, partially<br>☐No	☐Yes<br>☐No
If yes, what algorithm is used for screening this risk group?		
Homeless communities	☐Yes<br>☐Yes, partially<br>☐No	☐Yes<br>☐No
If yes, what algorithm is used for screening this risk group?		
Indigenous or tribal communities, or communities in remote or isolated areas	☐Yes<br>☐Yes, partially<br>☐No	☐Yes<br>☐No
If yes, what algorithm is used for screening this risk group?		
General population/community-wide screening (in areas with very high prevalence of TB)	☐Yes<br>☐Yes, partially<br>☐No	☐Yes<br>☐No
If yes, what algorithm is used for screening this risk group?		
Other (please describe risk groups and algorithms)	☐Yes<br>☐Yes, partially<br>☐No	☐Yes<br>☐No
For these community-based screening activities, what methods are used to reach people for screening? (check all that apply)<br>☐ Screening camps set up at community sites<br>☐ Mobile van-based screening<br>☐ Door-to-door screening<br>☐ Other (please describe)		

Are there any screening activities in the following ***mobile populations***?

At-Risk Groups	Systematic Screening *Implemented*	If screening is implemented, is chest X-ray used?
People in refugee camps	☐ Yes<br>☐ Yes, partially<br>☐ No	☐ Yes<br>☐ No
If yes, what algorithm is used for screening this risk group?		
Internally displaced persons	☐ Yes<br>☐ Yes, partially<br>☐ No	☐ Yes<br>☐ No
If yes, what algorithm is used for screening this risk group?		
Migrant workers	☐ Yes<br>☐ Yes, partially<br>☐ No	☐ Yes<br>☐ No
If yes, what algorithm is used for screening this risk group?		
Immigrants from settings with a high prevalence of TB	☐ Yes<br>☐ Yes, partially<br>☐ No	☐ Yes<br>☐ No
If yes, what algorithm is used for screening this risk group?		
People living in shelters	☐ Yes<br>☐ Yes, partially<br>☐ No	☐ Yes<br>☐ No
If yes, what algorithm is used for screening this risk group?		
Other (please describe risk groups and algorithms)	☐ Yes<br>☐ Yes, partially<br>☐ No	☐ Yes<br>☐ No
For screening activities in these mobile populations, what methods are used to reach people for screening? (check all that apply)<br>☐ Screening conducted at camps/shelters<br>☐ People referred to clinics for screening<br>☐ Mobile van-based screening<br>☐ Other (please describe)		

Are there any ***work-force based*** screening activities for the following populations?

Work-force Population	Systematic Screening *Implemented*	If screening is implemented, is chest X-ray used?
People working in TB or veterinary medicine laboratories	☐ Yes<br>☐ Yes, partially<br>☐ No	☐ Yes<br>☐ No
If yes, what algorithm is used for screening this risk group?		

Prison guards and other workers in penitentiary facilities	☐ Yes<br>☐ Yes, partially<br>☐ No	☐ Yes<br>☐ No
If yes, what algorithm is used for screening this risk group?		
Health-care workers	☐ Yes<br>☐ Yes, partially<br>☐ No	☐ Yes<br>☐ No
If yes, what algorithm is used for screening this risk group?		
Miners	☐ Yes<br>☐ Yes, partially<br>☐ No	☐ Yes<br>☐ No
If yes, what algorithm is used for screening this risk group?		
Military personnel	☐ Yes<br>☐ Yes, partially<br>☐ No	☐ Yes<br>☐ No
If yes, what algorithm is used for screening this risk group?		
Other (please describe risk groups and algorithms)	☐ Yes<br>☐ Yes, partially<br>☐ No	☐ Yes<br>☐ No

SECTION 6: Expansion of Screening for TB disease

Does your programme plan to expand your current TB disease screening activities?	☐ Yes - please describe how (which subgroups? which settings? which tools?):	
	☐ No - Please provide an explanation why not (budget / resources / personnel, etc):	
What might motivate your programme to further expand screening ?	☐ New evidence or modeling of effectiveness of screening<br>☐ New global guidelines or implementation guidance<br>☐ More funding<br>☐ More political will<br>☐ Other (please describe)	
For **planning new or expanded screening**, does your programme have any further information or documentation that could be shared with us?		☐ Yes<br>☐ No
If yes, please either describe here or attach supporting documents along with this completed survey in your reply e-mail:		

SECTION 7. Subclinical TB

With systematic screening persons with TB disease may be detected with microbiology and/or chest radiography, who do not report TB symptoms. These are sometimes referred to as subclinical TB:

Does your programme include such patients with your notified TB cases?	☐ Yes ☐ No
Does your programme have a definition of subclinical TB in your national TB guidelines or your surveillance guidance?	☐ Yes ☐ No
If **yes**, please include it here:	
Does your programme treat patients with subclinical TB?	☐ Yes ☐ No
If **yes**, please explain how:	

SECTION 8: Data on TB screening

Data systems

- What types of data are gathered for TB screening at the national level?
- How are the data collected, collated, reported?
- What systems are using for collecting, monitoring, analyzing data? (digital versus paper, connectivity of digital systems with the TB programme and wider health systems, etc.)
- How are screening data used at the national level?
    - What indicators related to TB screening are monitored?
    - What targets are set for these indicators and how are they established?
    - How are they used to inform national decision making regarding TB screening?

Data tables

Table 1. Total number of people systematically screened for TB disease and the cascade of screening data in the last three years: (Note that we ask about number screened for ***people living with HIV, contacts*** and ***persons in prisons*** below. If screening is only done in these 3 groups, skip this table)

	TB screening cascade data				Total TB disease reported in same jurisdiction/population (if available)	Total TB disease reported in country
	Total screened	Total screened positive	Total evaluated for TB disease	Total TB disease diagnosed		
2021						
2022						

2023						

Table 2. TB screening data for people living with HIV (can make separate tables for different subpopulations of PLHIV, eg people newly enrolling in ART versus people engaged in regular care vs children, etc. if data available)

	Total eligible (total PLHIV)	Total screened	Total screened positive	Total evaluated for TB disease	TB disease diagnosed
2021					
2022					
2023					

Table 3. TB screening data for contacts of TB patients

	Total eligible	Total screened	Total screened positive	Total evaluated for TB disease	TB disease detected
2021					
2022					
2023					

Table 4. TB screening data for prisoners

	Total eligible	Total screened	Total screened positive	Total evaluated for TB disease	TB disease detected
2021					
2022					
2023					

Table 5. TB screening data for facility-based screening (ICF)

	Total eligible	Total screened	Total screened positive	Total evaluated	TB disease detected

				for TB disease	
2021					
2022					
2023					

Table 6. TB screening data for community-based screening (ACF)

	Total eligible	Total screened	Total screened positive	Total evaluated for TB disease	TB disease detected
2021					
2022					
2023					

Additional tables can be added for other specific risk groups if data are available (e.g. miners, health workers, others)

The interview is completed – thank you!

2. Interview Guide for TB screening Funders

The following interview guide has been developed to gather information on funding support for systematic TB screening activities from major funders in high TB burden countries. The findings will be compiled into a written report and used as a supportive document for a WHO technical consultation on TB screening which will be held in Geneva in October 2024. Thank you in advance for your time.

- What types of activities are you supporting related to TB screening?

- How are decisions made with regard to what types of screening activities to support? How are screening activities prioritized? How are decisions regarding scaling up or scaling down screening activities made? How is the sustainability of TB screening considered?

- What level of funding are you providing for TB screening in the country? Are there other types of support provided for TB screening (e.g. in-kind support, equipment, supplies, etc.)?

- How do you evaluate the success of funding for TB screening?
    - What metrics are used to evaluate screening efforts?
    - How are the benchmarks set - how is success defined?

- Do you have reports from funded TB screening activities that you can share?

- How has your approach to TB screening changed in the last 3 years? Why?

- What are future plans with regard to TB screening activities?

3. Interview Guide for Subnational TB Programmes:

Survey of subnational translation of policies, implementation, and results of TB screening activities

The following interview guide has been developed to gather information on current tuberculosis (TB) disease systematic screening activities in subnational regions of high TB burden countries. The findings will be compiled into a written report and used as a supportive document for a WHO technical consultation on TB screening which will be held in Geneva in October 2024. Thank you in advance for your time

- Subnational level questions around translation of national policy, training, resource availability, M&E/surveillance

SECTION 1: General Information about TB screening policy and implementation

Brief questions to identify your programme.	
Date survey completed (dd/mm/yyyy):	
What is your name, and email address (in case clarification is needed)?	
Country	
Name of TB Programme/Institution	
What jurisdictions are covered by your region?	
What is the total population served by your region?	

- How are national policies for TB screening implemented in your region? Are they implemented as written or are they adapted, and why?

- Who are the partners implementing TB screening in your region? (include all that apply)
    - ☐ National health programmes (NTP, HIV, MCH, primary care/NCD screening)
    - ☐ Occupational health screening
    - ☐ Penitentiary screening
    - ☐ Implementing partners (INGOs, NGOs, local implementing partners, faith-based organizations, others as applicable)
        - Please specify
    - ☐ Private sector providers (if implementing screening)
    - ☐ Mining sector (if applicable)
    - ☐ Other (please specify):

- How are screening activities funded in your region?
    - ☐ Government (national) funded
    - ☐ Government (local) funded
    - ☐ Donor funded
    - ☐ Special projects
    - ☐ Not applicable
    - ☐ Other (please specify):

- Are screening services always provided free of charge or are there sometimes fees for patients?

- How is training prior to screening implementation conducted in your region? Who are the trainers?

Overall challenges, enablers

- What are the most challenging aspects of implementing screening in your region?

- What are the biggest challenges with implementing CXR-based screening (with or without computer-aided detection, or CAD)?

- What are enablers for screening implementation? What have you found essential to successful implementation of TB screening?

- In what areas of screening implementation is there a need for further research, guidance, technical support?

Section 2: Tools used for TB disease screening:

<table>
<tr><td colspan="2">Is chest X-ray (CXR) use recommended in your national guideline for the screening of TB disease?</td><td>☐ Yes
☐ No</td></tr>
<tr><td>Is CXR used in practice for the screening of TB disease in your region?</td><td colspan="2">☐ Yes always/regardless of symptoms
☐ Yes, sometimes
☐ Yes but only after symptom screening
☐ No
If CXR is not used systematically or at all, what are the reasons?
☐ Lack of availability of technology
☐ High cost
☐ Lack of expertise in interpretation
☐ Not the preferred screening approach
☐ Not determined necessary
☐ Other (please describe)</td></tr>
<tr><td colspan="3">Is computer-aided detection of TB-related abnormalities on chest radiography (CAD) use recommended in your national guideline for the screening of TB disease?
☐ Yes ☐ No</td></tr>
</table>

Is CAD used in practice for interpretation of CXR for the screening of TB disease **in your region**?	☐ Yes (if **yes**, please describe CAD solution providers)<br>☐ No<br>If yes, how was the threshold to be used for TB disease selected?<br>☐ Used the manufacturer's recommended threshold<br>☐ Pragmatic approach to threshold selection<br>☐ Conducted a calibration study (via operational research)<br>☐ Other (please describe)<br><br>If CAD is **not** used systematically or not at all, what are the reasons?<br>☐ High cost<br>☐ Lack of technical expertise<br>☐ Technological infrastructure (eg internet access, electricity)<br>☐ Shortage of chest x-ray capacity<br>☐ Not necessary (no shortage of human capacity for interpretation)<br>☐ Not approved in country<br>☐ Other (please describe)

Is use of **molecular WHO-recommended rapid diagnostic test (mWRDs**) recommended in your national guideline to screen people with HIV or other risk groups, regardless of symptoms? (i.e. specifically for *screening* for TB disease, and **not** diagnosis)	☐ Yes<br>☐ No

Are mWRDs used in practice for the **screening** of TB disease (not just for diagnosis) in your region?	☐ Yes, used for screening<br>☐ Not used for screening (may be used for diagnosis)<br><br>If they are **not** used what are the reasons?<br><br>☐ Not aware of option for use for screening<br>☐ Difficulties to implement test in the screening context<br>☐ Financially unfeasible<br>☐ Lack or shortage of mWRD infrastructure or supplies<br>☐ Lack of sample transportation infrastructure<br>☐ Lack of ability to store samples<br>☐ Other (please describe)

SECTION 3: TB DISEASE SCREENING ACTIVITIES IN THREE SPECIFIC HIGH RISK POPULATIONS

I. People living with HIV (PLHIV)

For **PLHIV**, is systematic screening recommended according to your national guideline?	☐ Yes<br>☐ No
For **PLHIV**, is systematic screening implemented in practice **in your region**?	☐ Yes<br>☐ Yes, partially implemented<br>☐ No
If **yes**, for **PLHIV,** which of the following algorithms are employed for screening? Please refer to **this link** if needed.	
☐ WHO-recommended four-symptom screen (W4SS) single screening algorithm<br>☐ C-reactive protein (CRP) single screening algorithm<br>☐ CXR single screening algorithm<br>☐ Parallel screening algorithm with W4SS and CRP<br>☐ Sequential positive screening algorithm with W4SS and CRP	

☐ Sequential negative screening algorithm with W4SS and CRP<br>☐ Parallel screening algorithm with W4SS and CXR<br>☐ Sequential positive screening algorithm with W4SS and CXR<br>☐ Sequential negative screening algorithm with W4SS and CXR<br>☐ mWRD single screening algorithm<br>☐ Other (please describe)
If yes, with what frequency are PLHIV screened?<br>☐ Once a year ☐ At every visit to a health center<br>☐ Other, or variable (please describe)

II. Household contacts and other close contacts of individuals with TB disease

For **close contacts**, is systematic screening for TB disease recommended according to your national guideline?	☐ Yes<br>☐ No
For **close contacts**, is systematic screening implemented in practice **in your region**?<br>*(If **yes**, please also complete next 3 questions)*	☐ Yes<br>☐ Yes, partially<br>☐ No
Is systematic screening implemented in all age groups? ☐ Yes ☐ No<br>If **no**, please specify what ages are screened: *(check all that apply)*<br>☐ Individuals aged ≥15 years ☐ Children aged 5-14 years ☐ Children aged <5 years	
For **close contacts,** which of the following algorithms are employed for screening? Please refer to **this link** if needed.	
☐ Screening with cough<br>☐ Parallel screening with cough and CXR<br>☐ Sequential positive serial screening with cough and CXR<br>☐ Sequential negative serial screening with cough and CXR<br>☐ Screening with any TB symptom<br>☐ Parallel screening with any TB symptom and CXR<br>☐ Sequential positive serial screening with any TB symptom and CXR<br>☐ Sequential negative serial screening with any TB symptom and CXR<br>☐ Screening with CXR followed by mWRD<br>☐ Screening with mWRD followed by diagnostic exam<br>☐ Other (please describe)	
For close contacts, what methods are used to reach contacts for screening? (check all that apply)<br>☐ Invite contacts to come to clinic for evaluation<br>☐ Visit the home to screen contacts<br>☐ Other(please describe)	

III. People in prisons and penitentiary institutions

For **incarcerated individuals**, is systematic screening for TB disease recommended according to your national guideline?	☐ Yes<br>☐ No
For **incarcerated individuals**, is systematic screening for TB disease implemented in practice **in your region**? *(If yes, please also complete next 3 questions)*	☐ Yes

	☐ Yes, partially<br>☐ No

For **incarcerated individuals,** which of the following algorithms are employed for screening? Please refer to **[this link](#)** if needed.
☐ Screening with cough<br>☐ Parallel screening with cough and CXR<br>☐ Sequential positive serial screening with cough and CXR<br>☐ Sequential negative serial screening with cough and CXR<br>☐ Screening with any TB symptom<br>☐ Parallel screening with any TB symptom and CXR<br>☐ Sequential positive serial screening with any TB symptom and CXR<br>☐ Sequential negative serial screening with any TB symptom and CXR<br>☐ Screening with CXR followed by mWRD<br>☐ Screening with mWRD followed by diagnostic exam<br>☐ Other (please describe)

SECTION 4: FACILITY BASED TB DISEASE SCREENING ACTIVITIES IN AT-RISK GROUPS

Please indicate if systematic screening for TB disease is performed in hospitals (outpatient or inpatient departments) or primary care centres, for persons with the following risk factors:

**At-risk Groups**	**Systematic Screening *Implemented***	**If screening is implemented, is chest X-ray used?**
People with chronic respiratory disease or lung conditions (including fibrotic lesions on CXR, previous treatment for TB, others)	☐ Yes<br>☐ Yes, partially<br>☐ No	☐ Yes<br>☐ No
People with diabetes mellitus	☐ Yes<br>☐ Yes, partially<br>☐ No	☐ Yes<br>☐ No
People who smoke (defined as those who have smoked 100 cigarettes in their lifetime and who currently smoke cigarettes)	☐ Yes<br>☐ Yes, partially<br>☐ No	☐ Yes<br>☐ No
People who are undernourished, or with a body mass index <18	☐ Yes<br>☐ Yes, partially<br>☐ No	☐ Yes<br>☐ No
People with alcohol or drug use disorder	☐ Yes<br>☐ Yes, partially<br>☐ No	☐ Yes<br>☐ No
General outpatients and inpatients (where prevalence of both TB and of TB risk factors is high)	☐ Yes<br>☐ Yes, partially<br>☐ No	☐ Yes<br>☐ No
Children other than contacts or HIV infection	☐ Yes<br>☐ Yes, partially<br>☐ No	☐ Yes<br>☐ No
Persons in mental health institutions or clinics	☐ Yes<br>☐ Yes, partially<br>☐ No	☐ Yes<br>☐ No

Persons in other residential facilities (long term care)	☐ Yes<br>☐ Yes, partially<br>☐ No	☐ Yes<br>☐ No
Other (please describe)	☐ Yes<br>☐ Yes, partially<br>☐ No	☐ Yes<br>☐ No

SECTION 5: OTHER LOCATION-BASED TB DISEASE SCREENING ACTIVITIES IN AT-RISK GROUPS

Are there any screening activities in ***community sites*** for the following population groups?

**At-Risk Groups**	**Systematic Screening *Implemented***	**If screening is implemented, is chest X-ray used?**
Poor urban (slum) communities	☐ Yes<br>☐ Yes, partially<br>☐ No	☐ Yes<br>☐ No
Homeless communities	☐ Yes<br>☐ Yes, partially<br>☐ No	☐ Yes<br>☐ No
Indigenous or tribal communities, or communities in remote or isolated areas	☐ Yes<br>☐ Yes, partially<br>☐ No	☐ Yes<br>☐ No
General population/community-wide screening (in areas with very high prevalence of TB)	☐ Yes<br>☐ Yes, partially<br>☐ No	☐ Yes<br>☐ No
Other (please describe)	☐ Yes<br>☐ Yes, partially<br>☐ No	☐ Yes<br>☐ No
For these community-based screening activities, what methods are used to reach people for screening? (check all that apply)<br>☐ Screening camps set up at community sites<br>☐ Mobile van-based screening<br>☐ Door-to-door screening<br>☐ Other (please describe)		

Are there any screening activities in the following ***mobile populations***?

**At-Risk Groups**	**Systematic Screening *Implemented***	**If screening is implemented, is chest X-ray used?**
People in refugee camps	☐ Yes<br>☐ Yes, partially<br>☐ No	☐ Yes<br>☐ No
Internally displaced persons	☐ Yes<br>☐ Yes, partially<br>☐ No	☐ Yes<br>☐ No

Migrant workers	☐ Yes<br>☐ Yes, partially<br>☐ No	☐ Yes<br>☐ No
Immigrants from settings with a high prevalence of TB	☐ Yes<br>☐ Yes, partially<br>☐ No	☐ Yes<br>☐ No
People living in shelters	☐ Yes<br>☐ Yes, partially<br>☐ No	☐ Yes<br>☐ No
Other (please describe)	☐ Yes<br>☐ Yes, partially<br>☐ No	☐ Yes<br>☐ No
For screening activities in these mobile populations, what methods are used to reach people for screening? (check all that apply)<br>☐ Screening conducted at camps/shelters<br>☐ People referred to clinics for screening<br>☐ Mobile van-based screening<br>☐ Other (please describe)		

Are there any ***work-force based*** screening activities for the following populations?

**Work-force Population**	**Systematic Screening *Implemented***	**If screening is implemented, is chest X-ray used?**
People working in TB or veterinary medicine laboratories	☐ Yes<br>☐ Yes, partially<br>☐ No	☐ Yes<br>☐ No
Prison guards and other workers in penitentiary facilities	☐ Yes<br>☐ Yes, partially<br>☐ No	☐ Yes<br>☐ No
Health-care workers	☐ Yes<br>☐ Yes, partially<br>☐ No	☐ Yes<br>☐ No
Miners	☐ Yes<br>☐ Yes, partially<br>☐ No	☐ Yes<br>☐ No
Military personnel	☐ Yes<br>☐ Yes, partially<br>☐ No	☐ Yes<br>☐ No
Other (please describe)	☐ Yes<br>☐ Yes, partially<br>☐ No	☐ Yes<br>☐ No

SECTION 8: Data on TB screening

Data systems

- What types of data are gathered for TB screening at the regional level?

- How are the data collected, collated, reported?

- What systems are using for collecting, monitoring, analyzing data? (digital versus paper, connectivity of digital systems with the TB programme and wider health systems, etc.)

- How are screening data used?
    - What indicators related to TB screening are monitored?
    - What targets are set for these indicators and how are they established?
    - How are they used to inform regional decision making regarding TB screening?

Data tables

Table 1. Total number of people systematically screened for TB disease and the cascade of screening data in the last three years: (Note that we ask about number screened for ***people living with HIV, contacts*** and ***persons in prisons*** below. If screening is only done in these 3 groups, skip this table)

	TB screening cascade data				Total TB disease reported in same jurisdiction/population (if available)	Total TB disease reported in region
	Total screened	Total screened positive	Total evaluated for TB disease	Total TB disease diagnosed		
2021						
2022						
2023						

Table 2. TB screening data for people living with HIV (can make separate tables for different subpopulations of PLHIV, eg people newly enrolling in ART versus people engaged in regular care vs children, etc. if data available)

	Total eligible (total PLHIV)	Total screened	Total screened positive	Total evaluated for TB disease	TB disease diagnosed
2021					

2022					
2023					

Table 3. TB screening data for contacts of TB patients

	Total eligible	Total screened	Total screened positive	Total evaluated for TB disease	TB disease diagnosed
2021					
2022					
2023					

Table 4. TB screening data for prisoners

	Total eligible	Total screened	Total screened positive	Total evaluated for TB disease	TB disease diagnosed
2021					
2022					
2023					

Table 5. TB screening data for facility-based screening (ICF)

	Total eligible	Total screened	Total screened positive	Total evaluated for TB disease	TB disease diagnosed
2021					
2022					
2023					

Table 6. TB screening data for community-based screening (ACF)

	Total eligible	Total screened	Total screened positive	Total evaluated for TB disease	TB disease diagnosed

2021					
2022					
2023					

Additional tables can be added for other specific risk groups if data are available (e.g. miners, health workers, others)

The survey is completed – thank you!

4. Interview Guide for Screening Implementation Sites

Country survey of policies, activities, and results of active case finding (Screening for TB disease)

The following interview guide has been developed to gather information on current tuberculosis (TB) disease systematic screening activities in key high TB burden countries. The findings will be compiled into a written report and used as a supportive document for a WHO technical consultation on TB screening which will be held in Geneva in October 2024. Information from sites where TB screening is being implemented will be essential to inform these discussions and future WHO guidance on this subject. Thank you in advance for your time

Section 1. Background

Brief questions to identify your organization.	
Date survey completed (dd/mm/yyyy):	
What is your name, and email address (in case clarification is needed)?	
Name of Organization	
What objectives or jurisdictions are covered by your programme?	
What is the total population served by your programme?	

- Can you describe your screening activities?
    - What risk groups do you screen?
    - What screening tools and algorithms do you use?
    - How is a TB diagnosis established or TPT initiated?
    - How is your target population reached through screening?
    - Is your TB screening activity integrated with any other type of health or social service provision?

- Who is providing funding for your screening activities?

- How are decisions made around what screening activities to conduct? Are they done in conjunction with NTP or other groups?

- Are national policies implemented as written in your screening activities (including risk groups and screening tools/algorithms) or are they modified? If so, how?

Section 2.Training and Implementation

Training

- How is training prior to screening implementation conducted? Who are the trainers?

- Do the people conducting the screening utilize any job aids or tools? (e.g. are screening protocols available on-site in any form, are mobile digital tools used to assist with implementation)?

Implementation

- How are people recruited and selected for screening? Is selection truly systematic or is there some element of preselection/clinical triage?

- For clinic-based or community site-based screening:
    - How are people enrolled in screening?
    - How are people retained in the screening process? Are people kept on-site until results of screening tests are received, in order to be sent for diagnostic evaluation or TPT, or are they called back once results are available?
    - How are those diagnosed through screening linked to care? Likewise for TPT

- For community-based screening activities:
    - How are people engaged in the screening process?
    - How are people retained in the screening process?
    - How are people diagnosed through screening linked to care? Likewise for TPT

- For home-based screening interventions:
    - How are home visits arranged and conducted? How many home visits are typically required to reach all people to be screened?
    - Are people transported to clinics for further evaluation, if necessary? How is follow-up done? How are those diagnosed through screening linked to care? Likewise for TPT

Laboratory

- How do screening activities impact the workload and workflow in laboratories?

- What confirmatory testing is used for people who screen positive?

Overall challenges, enablers

- What are the most challenging aspects of implementing screening?

- What are the biggest challenges with implementing CXR-based screening (with or without computer-aided detection, or CAD)?

- What are enablers for screening implementation? What have you found essential to successful implementation of TB screening?

- In what areas of screening implementation is there a need for further research, guidance, technical support?

Section 3. Data on TB screening

Data systems

- What types of data are gathered for screening?

- How is the data collected, collated, reported?

- What systems are using for collecting, monitoring, analyzing data? (digital versus paper, connectivity of digital systems with the TB programme and wider health systems, etc.)

- How are the screening data used at your implementation site?

    - What indicators related to TB screening are monitored?
    - What targets are set for these indicators and how are they established?
    - How are they used to inform decision making regarding TB screening?

Data tables below, if data are available. Please complete any tables that are applicable and delete the rest.

Table 1. Total number of people systematically screened for TB disease and the cascade of screening data in the last three years

	TB screening cascade data				Total TB disease reported in same jurisdiction/population (if available)	Total TB disease reported in country
	Total screened	Total screened positive	Total evaluated for TB disease	Total TB disease diagnosed		
2021						
2022						
2023						

Table 2. TB screening data for people living with HIV (can make separate tables for different subpopulations of PLHIV, eg people newly enrolling in ART versus people engaged in regular care vs children, etc. if data available)

	Total eligible	Total screened	Total screened positive	Total evaluated	TB disease diagnosed

	(total PLHIV)			for TB disease	
2021					
2022					
2023					

Table 3. TB screening data for contacts of TB patients

	Total eligible	Total screened	Total screened positive	Total evaluated for TB disease	TB disease detected
2021					
2022					
2023					

Table 4. TB screening data for prisoners

	Total eligible	Total screened	Total screened positive	Total evaluated for TB disease	TB disease detected
2021					
2022					
2023					

Table 5. TB screening data for facility-based screening (ICF)

	Total eligible	Total screened	Total screened positive	Total evaluated for TB disease	TB disease detected
2021					
2022					
2023					

Table 6. TB screening data for community-based screening (ACF)

	Total eligible	Total screened	Total screened positive	Total evaluated for TB disease	TB disease detected
2021					
2022					
2023					

Additional tables can be added for other specific risk groups if data are available (e.g. miners, health workers, others)

The interview is completed – thank you!
